# Priming iTBS for lower limb rehabilitation after stroke: Protocol for a randomized controlled trial on efficacy and neuroplasticity

**DOI:** 10.1371/journal.pone.0349578

**Published:** 2026-05-21

**Authors:** Zhipeng Pan, Jianxia Lu, Yue Sun, Yao Qian, Yi Shao, Chengchen Chen, Hongrui Liu, Jianchen Zhang, Zixuan Huang, Limin Sun, Kewei Yu, Ya Zhou, Hewei Wang, Bin Su

**Affiliations:** 1 School of Rehabilitation, Shandong University of Traditional Chinese Medicine, Jinan, Shandong, China; 2 School of Rehabilitation, Jiangsu Medical College, Yancheng, Jiangsu, China; 3 Department of Rehabilitation, Wuxi Central Rehabilitation Hospital, The Affiliated Mental Health Center of Jiangnan University, Wuxi, Jiangsu, China; 4 School of International Education, Jiangsu Medical College, Yancheng, Jiangsu, China; 5 Department of Rehabilitation, Huashan Hospital, Fudan University, Shanghai, China; 6 Department of Rehabilitation, Yancheng No. 1 People’s Hospital, Yancheng, Jiangsu, China; Hangzhou Normal University, CHINA

## Abstract

**Introduction:**

Repetitive transcranial magnetic stimulation (rTMS) is widely applied in treating motor dysfunction after stroke. Building on metaplasticity principle, continuous theta burst stimulation (cTBS) preconditioning enhances subsequent excitatory effects of intermittent theta burst stimulation (iTBS). This phenomenon is well-established for upper limbs in healthy populations, yet its application to the lower limbs remains underexplored, especially in stroke survivors. Guided by “central-peripheral-central” theory, this protocol investigates the synergistic effects of cTBS-preconditioned iTBS combined with lower limb robotic exoskeleton training for functional recovery and neuroplasticity after stroke.

**Methods and analysis:**

This single-blind, three-arm randomized controlled trial will assess the effects of priming iTBS combined with REX exoskeleton training in subacute stroke patients. Participants (n = 60) will be randomly assigned 1:1:1 to: (1) priming (cTBS + iTBS), (2) non-priming (sham cTBS + iTBS), or (3) sham stimulation (sham cTBS + sham iTBS). Stimulation targets lower-limb primary motor cortex (M1) using a double-cone coil (80% active motor threshold). All groups will receive REX exoskeleton training as conventional therapy to facilitate sensorimotor loop integration through proprioceptive feedback. The intervention lasts for 4 weeks, with assessments at baseline, mid-intervention, post-intervention, and 6-week follow-up. Primary outcome is the Fugl-Meyer Assessment of Lower Extremity (FMA-LE) score. Secondary outcomes include the Postural Assessment Scale for Stroke (PASS), Modified Barthel Index (MBI), and three-dimensional gait analysis, complemented by surface electromyography (sEMG) to assess muscle activation patterns and coordination during functional movements. The functional near-infrared spectroscopy (fNIRS) functional connectivity, motor evoked potential (MEP) indices, and electroencephalography (EEG) signals will be analyzed to explore the neural plasticity underlying the treatment effects. Data will be analyzed using mixed-design analysis of variance (ANOVA), and the study achieves 90% power to detect FMA-LE differences with 20 participants per group.

## Introduction

Lower limb motor impairment post-stroke presents a significant clinical challenge [1], driving demand for advanced neurorehabilitation strategies that leverage neuroplasticity [2]. Repetitive transcranial magnetic stimulation (rTMS), particularly time-efficient patterned protocols like theta-burst stimulation (TBS), hold promises for modulating motor recovery in stroke [3–5]. While intermittent TBS (iTBS) typically facilitates cortical excitation and continuous TBS (cTBS) suppresses it, recent work reveals that synaptic history crucially influences neuroplastic responses through metaplasticity.

Metaplasticity, often described as the plasticity of synaptic plasticity, serves as a homeostatic mechanism by which prior neural activity bidirectionally adjusts the threshold for subsequent induction of plasticity [6,7]. Priming strategies leverage this phenomenon; for example, cTBS preconditioning induces metaplastic stabilization that enhances neuroplastic responses to subsequent iTBS, boosting long-term potentiation-like effects in the human motor cortex [8,9]. This priming effect has been demonstrated in the upper-limb M1 of both healthy individuals and stroke patients, where cTBS-iTBS sequences enhance motor recovery more effectively than iTBS alone [5,10]. Mechanistically, cTBS lowers the threshold for ensuing facilitatory plasticity by modulating N-methyl-D-aspartate receptor trafficking and triggering intracellular cascades that prime synapses for iTBS-induced potentiation [6,11].

Critically, evidence for such metaplastic effects comes almost entirely from upper-limb studies [5,12,13]. Zhang et al. randomized 42 patients into three groups receiving 10 sessions of priming iTBS (with cTBS), non-priming iTBS, or sham stimulation, followed by robot-assisted training. Priming iTBS led to greater improvement in upper limb function compared to the other groups. It also enhanced ipsilateral hemisphere high-beta band sensorimotor event-related desynchronization during mirror visual feedback, suggesting increased responsiveness of ipsilateral sensorimotor areas to sensory stimuli, potentially supporting motor learning and recovery [10]. Although priming protocols effectively enhance cortical excitability and motor recovery in upper limb rehabilitation after stroke, studies on lower limb applications remain notably limited. The translational applicability of cTBS-iTBS priming to leg motor areas thus remains underexplored. Our recent work directly addresses this knowledge gap by providing the first neurophysiological evidence that cTBS preconditioning enhances iTBS-induced corticomotor excitability in the lower-limb motor cortex of healthy adults [14]. This preclinical study demonstrates the feasibility of targeting metaplasticity in lower limb motor regions and elucidates the underlying mechanisms, thereby supporting the rationale for designing a clinical trial of cTBS-primed iTBS in post-stroke gait and balance rehabilitation.

During highly repetitive, physiological walking training, lower-limb exoskeletons deliver continuous, coordinated, and real-time proprioceptive and tactile sensory inputs to the affected limb, which robustly activates the sensorimotor cortex. [15,16]. Additionally, by providing precise mechanical assistance, exoskeletons guide the paretic limb through a near-normal gait cycle, effectively suppressing abnormal compensatory movement patterns and spasticity [17,18]. Furthermore, the positive psychological feedback and sense of achievement associated with regaining mobility significantly enhance patient motivation and engagement, thereby facilitating neuroplasticity and neural recovery.. Our team’s previous study [19] explored the impact of REX exoskeleton rehabilitation robot training on balance and lower limb function in patients with subacute stroke. Although the results showed that the REX exoskeleton rehabilitation robot has significant potential in promoting early recovery of balance and motor function in patients with subacute stroke, the small sample size limits the generalizability of the findings. In the future, it will be necessary to increase the sample size, conduct randomized controlled studies, and perform follow-up evaluations to verify the current research findings. Moreover, existing studies have mostly focused on the single-modal application of lower limb rehabilitation robots, and the application of their synergistic intervention with iTBS in lower limb rehabilitation after stroke has not been reported yet.

Existing studies have mostly focused on TMS metrics before and after rTMS intervention and found that it can improve lower limb function in stroke patients, accompanied by beneficial changes in cortical excitability [20,21], but the TMS ratings are limited to the excitability of individual brain regions or some inter-brain interactions, and it is difficult to comprehensively reflect the extensive structural and functional reorganization of the brain regions after stroke [22–25].Several studies have explored the mechanisms of rTMS using neuroimaging: Juan et al.’s RCT [26] showed that both HF-rTMS and LF-rTMS improved upper limb function in stroke patients, with a significant positive correlation between the activation level of the lesioned hemisphere and motor function. Jing et al. [27] found that rTMS could enhance interhemispheric functional connectivity (FC) and reduce intra-hemispheric FC in the lesioned hemisphere, but the study had a small sample size and lacked a randomized controlled design. Guo et al. [28] demonstrated that improved FC between the affected M1 and the contralateral premotor cortex was significantly correlated with enhanced neurological function, and the HF-rTMS group showed more significant therapeutic effects and greater increases in the abovementioned FC values. However, most of the studies are cross-sectional studies and lack longitudinal data; many of them do not have a control group, which makes it difficult to clarify the effect of rTMS alone; and there is a lack of studies on the neural mechanisms of rTMS and iTBS on the rehabilitation of lower limb motor function in stroke patients.

This randomized controlled trial aims to investigate the efficacy of priming iTBS combined with REX exoskeleton robot training in the rehabilitation of lower limb motor function in stroke patients. Additionally, longitudinal assessments using functional near-infrared spectroscopy (fNIRS), motor-evoked potentials (MEP), and three-dimensional gait analysis will be conducted before and after the intervention to explore the related neuroplasticity mechanisms.

We hypothesize that a 4-week priming iTBS plus Robot-Assisted Training intervention will yield significant improvement when compared to the control group. Additionally, we conjecture such functional improvements to correlate positively with two key neurophysiological changes: increased excitability of the ipsilesional primary motor cortex (M1) and enhanced functional connectivity within the sensorimotor motor networks. Furthermore, we anticipate the therapeutic benefits of this combined intervention will persist at the 6-week follow-up.

## Materials and methods

### Study design

**Fig 1:**
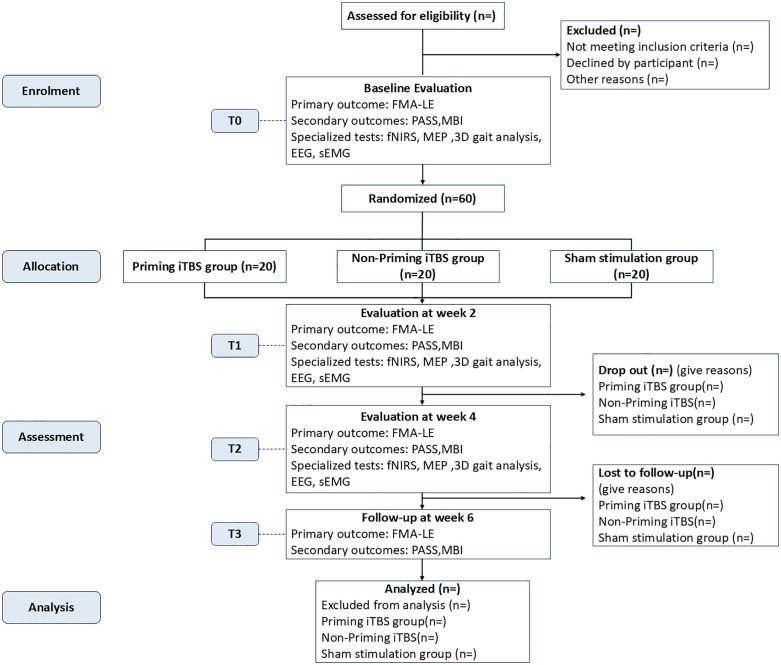
Flow diagram of the study design.

This study is a participant and outcome assessor-blinded, parallel-group, randomized controlled trial (RCT). Eligible participants will be randomly allocated to either the priming iTBS group (cTBS + iTBS), the non-priming iTBS group (sham cTBS + iTBS), or the sham stimulation group (sham cTBS + sham iTBS) in a 1:1:1 ratio. All groups will receive REX exoskeleton training as conventional therapy to facilitate sensorimotor loop integration through proprioceptive feedback. The intervention period will last for 4 weeks (5 days of intervention per week), with outcome assessments conducted at baseline (T0), midway at 2 weeks (T1), and immediately post-intervention at 4 weeks (T2), and a follow-up assessment will be conducted at 6 weeks (T3). This study is designed as a superiority trial. The trial will be conducted at the Department of Rehabilitation Medicine, Yancheng NO.1 People’s Hospital, Yancheng, Jiangsu, China. Fig 1 and Table 1 outline the trial procedures in detail.

**Table 1:** Schedule of enrolment, interventions, and assessments.

STUDY PERIOD	Enrolment	Allocation	Post-allocation			Follow-up
**TIMEPOINT**	**0**	**0**	**First intervention**	**Week 2**	**Week 4**	**Week 6**
**ENROLMENT**						
Eligibility screen	×					
Informed consent	×					
Randomization		×				
**INTERVENTION**						
Priming iTBS group			×	×	×	
Non-Priming iTBS group			×	×	×	
Sham stimulation group			×	×	×	
**ASSESSMENTS**						
**Primary outcome**						
FMA-LE	×			×	×	×
**Secondary outcomes**						
PASS	×			×	×	×
MBI	×			×	×	×
**Specialized tests**						
fNIRS	×			×	×	
EEG	×			×	×	
sEMG	×			×	×	
3D gait analysis	×			×	×	
MEP	×			×	×	
**Safety monitoring**						
Adverse events			×	×	×	×

iTBS, intermittent theta burst stimulation; FMA-LE, Fugl-Meyer Assessment of Lower Extremity; PASS, Postural Assessment Scale for Stroke; MBI, Modified Barthel Index; fNIRS, functional near-infrared spectroscopy; EEG, Electroencephalography; sEMG, Surface Electromyography; MEP, motor evoked potential.

This study has been approved by the Ethics Committee of Yancheng NO.1 People’s Hospital (approval number: 2025-K-184) and registered at the Chinese Clinical Trial Registry (ChiCTR2500110279). All participants will provide informed consent directly.The timeline for this study is as follows: recruitment of participants will commence on 01/02/2026 and is expected to be completed by 01/08/2026.; data collection is projected to conclude by 01/11/2026, with the aim of obtaining preliminary results by 01/12/2026.This study protocol will adhere to the principles of Helsinki and the Standard Protocol Items for Randomized Trials (SPIRIT) statement, and the checklist of this protocol is provided in the relevant supplementary materials (S1 File). Enrolling 60 participants within 6 months is feasible, supported by the approximately 500 annual stroke admissions at Yancheng NO.1 People’s Hospital, which ensures a sufficient candidate pool.

### Patient recruitment

Participants will be recruited from inpatient and outpatient stroke rehabilitation wards at Yancheng NO.1 People’s Hospital. Recruitment strategies include: (1) Displaying study posters and distributing flyers in relevant clinical areas. (2) Regular presentations about the trial to clinical staff (physicians, therapists) to facilitate patient referrals. (3) Screening of hospital admission records for potentially eligible patients by the research coordinator. An independent research coordinator will screen individuals for eligibility and obtain written informed consent prior to enrolment.

### Inclusion criteria

(1) Stroke diagnosed by CT or MRI. (2) Age 40–80 years old. (3) Stable condition, first-ever stroke (patients with a history of recurrent strokes will be explicitly excluded), duration 1–3 months. (4) Able to walk independently or with assistance for more than 10m, Brunnstrom stage III-V lower limb. (5) Cognitive clarity, ability to follow simple verbal commands or instructions, MoCA ≥ 26. (6) Informed consent signed by the patient or his/her relatives. (7) No history of epilepsy. (8) No contraindications to TMS examination.

### Exclusion criteria

(1) Individuals with relevant contraindications to TMS (pacemakers, intracranial implants, implanted drug pumps etc.). (2) Significant speech, attention, auditory, visual (lateral neglect, hemianopsia, etc.), intellectual, psychiatric or cognitive dysfunction. (3) Prior history of organic brain disease, neuropsychiatric history, drug abuse and alcoholism. (4) Patients with severe failure of vital organs (e.g., heart, lung, liver, kidney). (5) Patients with pre-existing lower limb motor dysfunction prior to the stroke. (6) Those who are currently participating in other clinical trials. (7) Those who cannot co-operate with the functional assessment and have poor compliance. (8) Inability to induce a TMS response in the tibialis anterior muscle prior to formal testing. (9) Pregnancy. (10) Use of drugs that affect cortical excitability (e.g., benzodiazepines, baclofen, antiepileptics, certain antidepressants) that cannot be safely tapered or washed out prior to study participation.

### Randomization and blinding

A total of sixty eligible stroke patients will be enrolled in this study. Following the baseline assessment, a computer-generated randomization sequence will be used to assign all participants in a 1:1:1 ratio to one of three parallel intervention groups: the priming iTBS group (n = 20), the non-priming iTBS group (n = 20), or the sham stimulation group (n = 20). The allocation sequence will be concealed from the researchers enrolling participants. After a participant provides informed consent and all baseline assessments (T0) are completed, the research coordinator will access the central web-based system. The system will then reveal the group allocation only to the unblinded physical therapist responsible for administering the intervention. This ensures that outcome assessors and participants remain blinded.

Physiotherapists and researchers delivering the conventional interventions will have access to the patient’s corresponding intervention group number through the system but will not be blinded to the specific intervention type. Blinding will be implemented for participants and outcome assessors throughout the trial. Blinding will be maintained until the end of the trial, after data locking, at which point unblinding will be performed.

Emergency unblinding will be permitted only in the event of a serious adverse event (SAE) where knowledge of the participant’s allocation is deemed necessary for their medical management. The request for unblinding will be made by the treating physician to the principal investigator, who will then access the randomization system. All instances of unblinding will be documented and reported to the Ethics Committee.

### Conventional rehabilitation protocol

All participants in the three groups will receive a standardized conventional rehabilitation regimen in addition to their respective trial interventions. This comprehensive foundational therapy is tailored to the individual’s needs but consistently includes the following core components:

**Physical Therapy:** Sessions target the improvement of gross motor functions, including bed mobility, transfers (e.g., from bed to chair), balance control in sitting and standing, and gait training. A key focus is placed on strength training for major muscle groups of the upper and lower limbs, utilizing modalities such as resistance bands, free weights, and weight-bearing exercises to address hemiparetic weakness. Additionally, therapists employ techniques to mitigate spasticity, facilitate normal movement patterns, and improve joint range of motion and flexibility.

**Occupational Therapy:** Interventions are designed to promote independence in Activities of Daily Living (ADLs), such as feeding, grooming, dressing, and bathing. Therapists also address upper limb function through targeted exercises for fine motor skills, coordination, and dexterity.

Furthermore, all patients will undergo a standardized robot-assisted training regimen utilizing the REX self-balancing exoskeleton rehabilitation robot (Rex Bionics Ltd., New Zealand). All robot-assisted training sessions are conducted after the TBS intervention. The interval between them should be minimized (less than 15 minutes). Each daily 30-minute session comprises three distinct 10-minute programs:

**Program 1: Standing Activity Training:** This component focuses on improving trunk control and upper-lower limb coordination during stable standing, exemplified by tasks such as targeted reaching.

**Program 2: Resistance Training with Elastic Bands:** Employing Proprioceptive Neuromuscular Facilitation (PNF) patterns, this program is designed to facilitate affected lower limb extension and enhance muscular strength and coordination.

**Program 3: Lower Limb Functional Training:** This segment aims to improve functional strength, balance, and mobility through exercises including single-leg weight-bearing, lateral stepping, and squats.

**Fig 2:**
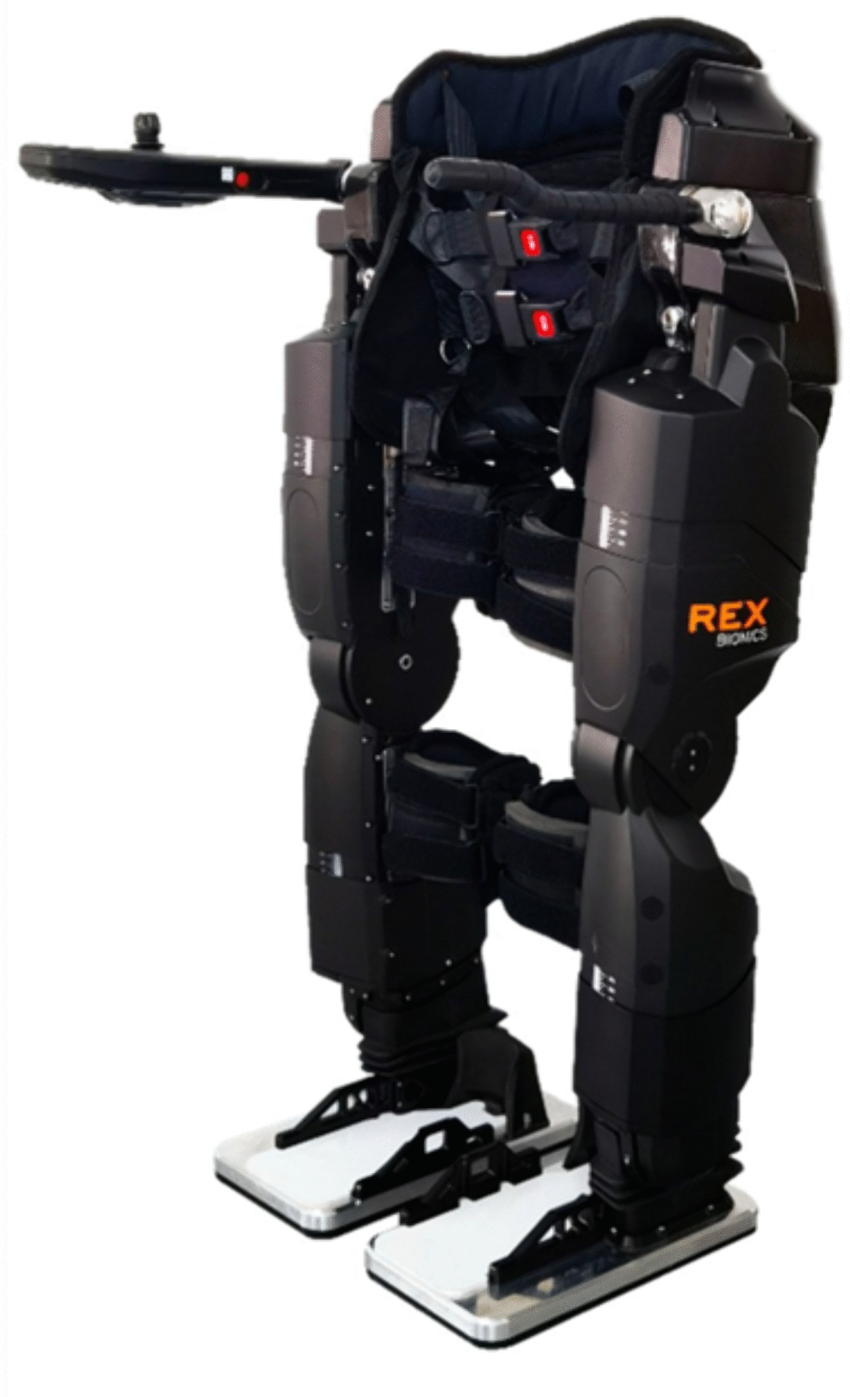
REX exoskeleton rehabilitation robot.

All robot-assisted training is supervised by experienced physical therapists. They dynamically adjust the level of robotic support and task difficulty based on real-time assessment of patient performance and tolerance, while providing concurrent verbal feedback. Detailed operational procedures, parameters, and monitoring protocols are rigorously specified in the study’s Standard Operating Procedure (SOP) (S2 File). Fig 2 shows the REX exoskeleton robot device and relevant treatment content.

### iTBS intervention protocol

Prior to each robot-assisted training session, the iTBS intervention will be administered using the Wuhan Yiruidian paired transcranial magnetic therapy device (model: N50000) with its matching double-cone coil. The stimulation will target the “hotspot” within the lower limb M1 area. This site will be determined by identifying the location that elicits the maximum motor evoked potential (MEP) amplitude in the tibialis anterior muscle, with the resting motor threshold (rMT) tested to assist in localization.

The intervention protocol will consist of two sequential parts: priming (using cTBS) followed by excitation (iTBS). The cTBS segment will deliver 600 pulses over approximately 40 seconds, using 200 clusters of uninterrupted stimulation at a 50 Hz pulse frequency and a 5 Hz repetition frequency. Subsequently, the iTBS segment will be administered. It will consist of a 2-second train of 3 pulses at 50 Hz, followed by an 8-second rest period. This 10-second cycle will be repeated 20 times, resulting in a total of 600 pulses over approximately 192 seconds. All stimulus intensities will be set at 80% of the individual’s active motor threshold (aMT), which is defined as the minimum intensity required to elicit at least 5 out of 10 peak-to-peak MEPs exceeding 200 μV in the tibialis anterior muscle.

For sham stimulation, the same double-cone coil used in the active condition will be employed. It will be configured to operate in a special sham mode, which mimics the audible “clicking” sound of active transcranial magnetic stimulation without delivering a clinically significant magnetic field to the scalp. The coil will be placed at the identical scalp position and held at the same angle as during active stimulation, using the same holder to ensure consistent positioning and blinding. This setup will replicate the acoustic characteristics of real TBS, generating a similar loud sound without delivering effective stimulation, thereby ensuring participant blindness to the intervention. The specific training operation details will be shown in the Standard Operating Procedure (SOP).

### Outcome measures

Assessments will be conducted at baseline (T0), 2 weeks (T1),4 weeks (T2) after intervention, and 6 weeks of follow-up (T3). Outcome measures will be collected by trained assessors blinded to group allocation. Assessors will have at least 5 years of clinical experience and receive specific training on standardized administration of all assessment tools prior to the trial start.

### Primary outcome

The primary outcome is the change in the Fugl-Meyer Assessment of Lower Extremity (FMA-LE) score from baseline (T0) to post-intervention (T2, 4 weeks). The FMA-LE quantifies the impairment and recovery of lower extremity motor function by systematically evaluating the dimensions of reflexes, synergistic movements, and coordination. The FMA-LE is scored on a 3-point ordinal scale: 0 for severe dysfunction, 1 for partial dysfunction, and 2 for no dysfunction, with a maximum total score of 34 points. With good reliability and validity, the FMA-LE has been widely recognized in clinical practice.

### Secondary outcomes

#### Clinical scales.

**Postural Assessment Scale for Stroke (PASS):** The secondary outcome is the change in the Postural Assessment Scale for Stroke (PASS) total score from baseline (T0) to post-intervention (T2, 4 weeks). The PASS is a postural control assessment tool designed specifically for stroke patients. The PASS consists of 12 items, each of which is divided into 4 grades (0–3) according to the patient’s ability to complete them, with higher scores indicating better postural control. It covers static postural maintenance, dynamic center of gravity shift, body position shift and anti-interference ability, and is a specific tool for postural control assessment in stroke patients.

**Modified Barthel Index (MBI):** The change in the MBI score from baseline (T0) to post-intervention (T2, 4 weeks) will be a secondary outcome. The Modified Barthel Index (MBI) will reflect the functional status of a patient by quantifying their level of independence in basic self-care, with the MBI focusing on “Activities of Daily Living” (ADL). It consists of 10 items covering three categories: self-care, mobility, and control, with a total score ranging from 0–100, with higher scores indicating better ADL function.

### Neurophysiological and biomechanical outcomes

**Fig 3:**
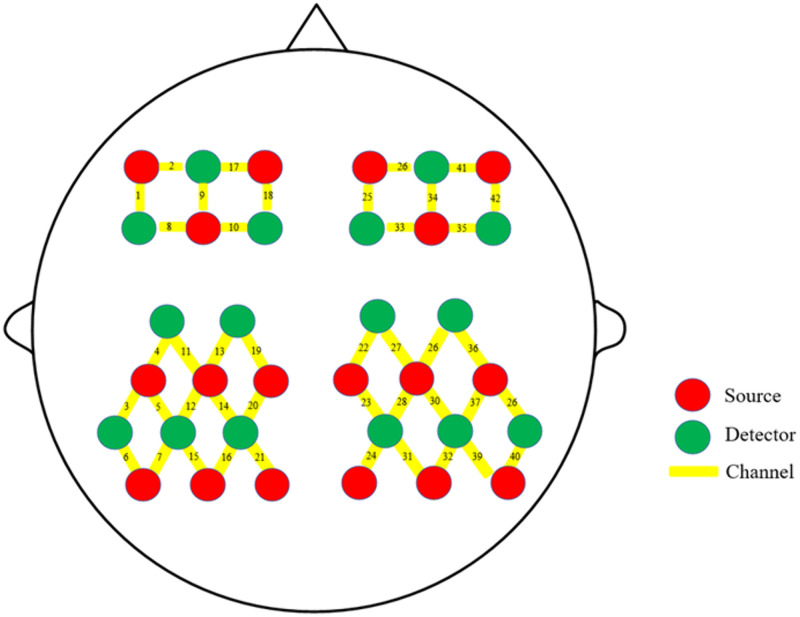
Layout of the fNIRS optodes and measurement channels.

Functional near-infrared spectroscopy (fNIRS) tests will be performed using the NirSmart-3000B device (Danyang Huichuang Medical Equipment Co., Ltd., China) to observe changes in the concentrations of oxygenated hemoglobin (HbO) and deoxygenated hemoglobin (HbR) in various brain regions. The fNIRS system consists of 16 light sources and 18 detectors (resulting in 40 active channels) with dual wavelength detection at 730nm and 850nm and a sampling frequency set to 11 Hz. Assessments will be conducted in baseline (T0), mid-intervention (T1, 2 weeks), and (T2, 4 weeks). Prior to fNIRS data acquisition, participants will remain in a quiet and relaxed state for approximately 1 minute to ensure a stable hemodynamic baseline. Subsequently, an 8-minute resting-state fNIRS scan will be conducted with participants maintaining a wakeful, motionless condition. Fig 3 shows the layout of the fNIRS system.

**Fig 4:**
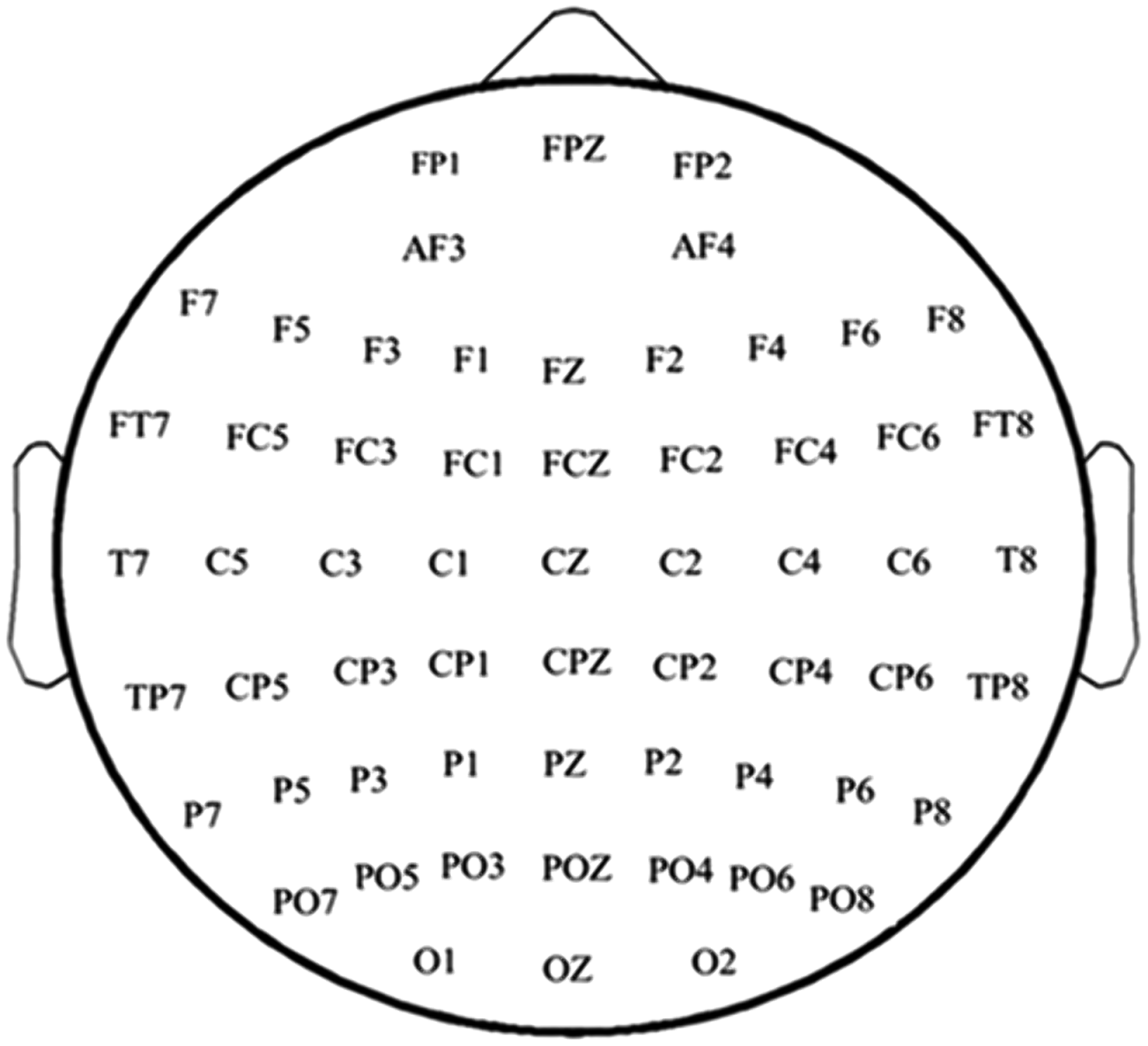
Configuration of the high-density EEG electrode system.

Electroencephalography signals will be acquired using a high-density 64-channel system (GES 400, EGI, USA). Electrodes will be arranged according to the international 10–20 system, with Cz as the reference, to record scalp EEG from 64 channels along with signals from bilateral mastoids. The system specifications (input impedance ≥1000 MΩ, sampling rate ≥8 kHz per channel, and input noise ≤1 μV) will ensure the capture of high-fidelity data. These signals will be analyzed to investigate treatment-induced changes in cortical activation and functional network dynamics as neural correlates of motor recovery. Prior to EEG signal acquisition, participants will remain in a quiet and relaxed state for approximately 1 minute to ensure a wakeful, relaxed, and motionless baseline condition. Subsequently, a 5-minute EEG recording will be conducted under eyes-closed resting conditions. Assessments will be conducted at baseline (T0), mid-intervention (T1, 2 weeks), post-intervention (T2, 4 weeks). Fig 4 shows the layout of the EEG system.

Gait data collection was performed using the Qualisys three-dimensional motion capture system. This system operates at a frequency of 100 Hz to accurately measure the three-dimensional coordinates of reflective markers placed at specific anatomical locations within the coronal, sagittal, and horizontal planes, thereby recording spatiotemporal and kinematic parameters that describe the body’s spatial movement during walking. Equipped with three-dimensional motion sensing and adaptive technology, the system utilizes computer learning algorithms to automatically track patients’ step frequency and gait characteristics, enabling precise detection of multi-dimensional angular changes. During walking tests, it automatically collects and analyzes basic gait parameters and continuously records usage data after a single power-on, including information such as the number of steps and time per step. Assessments were conducted at baseline (T0), mid-intervention (T1, week 2), and post-intervention (T2, week 4).

Surface electromyography signals will be recorded using a dedicated system (DELSYS, Trigno™, Wireless Biofeedback System, USA) from four lower limb target muscles (rectus femoris, biceps femoris, tibialis anterior, and gastrocnemius) to evaluate the effects of different intervention methods. Assessments will be conducted at baseline (T0), mid-intervention (T1, 2 weeks), post-intervention (T2, 4 weeks).

MEP assessment will be performed using a transcranial magnetic therapy instrument (Wuhan Iridium, N50000) and a biconical coil (VCZ001). DELSYS surface EMG electrodes will be placed on the affected tibialis anterior muscle (sampling frequency 2000 Hz), and the “hot spot” of the lower limb M1 zone will be localized based on the 10–20 EEG system (with AP direction current stimulation). The rMT and aMT will be measured, then stimulation will be delivered with 130% rMT intensity (interval ≥ 10s). A minimum of 15 valid MEP trials will be acquired at 130% rMT to record amplitude and latency. If recruitment curves (RC) are assessed, stimulus intensities will span 80–150% rMT (5 trials per 10% step) to derive RC parameters including slope. Muscle rest state (baseline RMS < 50 μV) and waveform stability (biphasic morphology, latency variability <10%) will be continuously monitored to ensure data quality. Assessments will be conducted at baseline (T0), mid-intervention (T1, 2 weeks), post-intervention (T2, 4 weeks).

### Statistical analysis

#### Sample size.

It was assumed that the FMA-LE scale endpoint of the priming iTBS group was 3 points higher than that of the non-pretreated iTBS group, and the combined standard deviation of all groups was 2.5 points. The sample size was calculated based on the results of existing studies and clinical experience [29], and at the statistically significant level of α = 0.05, to achieve 90% statistical validity, taking into account the 20% loss-to-follow-up rate, a minimum of 20 patients were required to be enrolled in each group, for a total of 60 patients, as calculated by the PASS 15 software.

### Analysis of clinical data

The statistical analyses will be performed using SPSS 22.0 software. Demographic and baseline characteristics will be compared by one-way analysis of variance (ANOVA) or Fisher’s test. Prior to formal analysis, the normality of data distribution will be assessed using the Shapiro-Wilk test. A mixed-design ANOVA was selected as the primary analytical framework because it robustly accommodates both the between-subject factor (the three parallel intervention groups) and the within-subject factor (repeated measures across four time points: T0, T1, T2, T3). This approach allows for the simultaneous evaluation of main effects and the crucial Group × Time interactions, which are essential for determining whether the treatment trajectories differ among the groups.

The significance level will be initially set at *p* < 0.05. If any significant Group × Time interaction effects are found, pairwise comparisons will be performed using paired t-tests, and the changes from baseline will be compared with a Bonferroni-corrected threshold of 0.017 (for 3 comparisons) to control Type I error in post-hoc testing of the primary outcome.

Furthermore, to control the overall false positive rate resulting from the evaluation of multiple secondary clinical outcomes (e.g., PASS, MBI, gait parameters) and high-dimensional neurophysiological analyses (e.g., fNIRS channels, EEG bands, MEP indices), the False Discovery Rate (FDR) correction (via the Benjamini-Hochberg procedure) will be applied to the resulting *p*-values across these secondary analyses.

### Analysis of neurophysiological and biomechanical data

The raw fNIRS data will be processed and analyzed using the Homer software package. The preprocessing pipeline begins with signal quality inspection and channel exclusion, in which channels with low signal-to-noise ratio or signal loss are flagged and excluded from further analysis. The raw light intensity data are then converted into optical density (OD) units, followed by motion artifact correction using algorithms such as spline interpolation or principal component analysis (PCA). A band-pass filter (0.01–0.2 Hz) is applied to remove low-frequency drift and high-frequency physiological noise (e.g., cardiac and respiratory cycles). Subsequently, the modified Beer–Lambert law is employed to derive time-series changes in oxygenated hemoglobin (HbO₂) and deoxygenated hemoglobin (HbR) concentrations.

Following preprocessing, regional brain activity will be assessed using the fractional amplitude of low-frequency fluctuations (fALFF) to quantify spontaneous neural activity at each channel. To evaluate functional integration within motor-related cortical networks, functional connectivity (FC) will be analyzed based on resting-state HbO₂ signals. Pearson correlation coefficients (r) will be computed between all pairs of channels and then converted to Z-scores via Fisher’s Z transformation to meet parametric assumptions. The resulting Z-scores will be averaged within and between four predefined regions of interest (ROIs)—PMC, SMA, DLPFC, and SMC—to generate ROI-level FC indicators. Between-group differences in FC will be tested using independent samples t-tests, with statistical significance set at p < 0.05.

To minimize the impact of noise and artifacts on data quality, all raw signals will be preprocessed using the EEGLAB toolbox in the MATLAB environment. The preprocessing pipeline includes band-pass filtering and independent component analysis (ICA) to effectively identify and remove physiological artifacts such as ocular and cardiac interference. Following this, a continuous 2-minute high-quality data segment free of significant artifacts will be extracted from each recording for subsequent spectral analysis.

This study focuses on alpha-band (8–13 Hz) activity in brain regions associated with motor function. Based on fast Fourier transform (FFT), the absolute power values within the alpha frequency band will be calculated for the following electrode sites: C3, C4, FC1, FC2, FCz, FC3, and FC4. These values will serve as core EEG metrics. Finally, correlations between the absolute alpha power in these regions and scores from clinical scales will be analyzed to explore the relationship between resting-state brain activity patterns and behavioral performance.

The efficacy of different intervention methods on lower limb muscle functional recovery will be evaluated by analyzing two key sEMG parameters: the Root Mean Square (RMS) and the Median Frequency (MF). In the acquired signals, the Root Mean Square (RMS), as a reliable parameter in the time-domain analysis, reflects the overall level of electrical activity of motor units during muscle contraction. Its value is associated with the recruitment of motor units and the synchronization of discharges, determines the variation in EMG amplitude, and is often used as a quantitative indicator of the force/torque generated by the muscle. On the other hand, the Median Frequency (MF) is a key parameter in the frequency-domain analysis, referring to the frequency value that bisects the area of the power spectrum of the EMG signal. It reflects the median level of muscle fiber discharge rates and is commonly used to assess local muscle fatigue and the mobilization of different muscle fiber types.

Electrodes will be positioned per SENIAM guidelines [30]: the active electrode centered 3 cm superior to the midpoint between the medial malleolus and tibial tuberosity over the tibialis anterior muscle belly, and a ground electrode on the medial malleolus. The biconical coil will be held tangential to the scalp, handle posteriorly directed at a 45° angle to the midline [31].

The “hot spot” (optimal scalp position for eliciting TA MEPs) will be determined using the 10–20 EEG system as a guide and confirmed by eliciting consistent MEPs with anterior-posterior directed current.

The resting motor threshold (rMT) is defined as the minimum stimulus intensity required to elicit motor-evoked potentials (MEPs) with a peak-to-peak amplitude ≥50 μV in at least 5 out of 10 consecutive stimuli. This is determined using a staircase procedure, starting at 30% of the maximum stimulator output and increasing in 1% increments [32]. The active motor threshold (aMT) is determined using the same criteria during voluntary muscle contraction. All procedures are performed under continuous electromyography (EMG) monitoring to ensure the tibialis anterior muscle remains at rest (root mean square [RMS] value <50 μV during baseline periods), with participants maintaining muscle relaxation aided by visual feedback.

Basic MEP parameters are acquired using a stimulus intensity of 130% rMT, with an inter-stimulus interval ≥10 seconds [33]. A minimum of 15 valid MEPs are obtained per set. If continuous stimulation is applied, inter-train intervals are dynamically adjusted based on heart rate variability, with a mandatory rest period of ≥1 minute enforced after every 10 stimuli. Optionally, a recruitment curve (RC) protocol may be performed: stimulus intensity is incrementally increased from 80% to 150% rMT in 10% steps, with 5 stimuli delivered at each intensity level (inter-stimulus interval ≥10 seconds) [34].

### Safety

Adverse events to be observed in this trial will include: hearing problems, local pain, muscle twitching, joint soreness, muscle fatigue, dizziness, and seizures. Seizures are the most severe acute adverse effect of iTBS, with improper parameter settings (e.g., excessive intensity relative to individual motor thresholds) increasing risk, as noted in safety guidelines. Joint soreness and muscle fatigue are more common in early robot-assisted training due to lower limb movement adaptation. Other side effects are generally mild and temporary, subsiding after short rest.

Our team will be fully prepared with an emergency area in the rehabilitation room to handle incidents. Immediate measures will be taken promptly if adverse events occur, with detailed documentation throughout the 4-week intervention.

### Patient and public involvement

Stroke patients in this study will participate as participants. No additional patients or members of the public will be involved in the study design, recruitment, or data analysis phases.

### Data management

Collected data will be entered into case report forms (CRF) and synchronized to the electronic database. Participants will be identified by unique numbers to ensure privacy and security. Paper documents will be stored in locked cabinets, and electronic data will be stored in password-protected computers. Original datasets will only be accessible to the principal investigator and relevant co-investigators of this study, and will be used solely for research purposes; all operations will strictly comply with medical data confidentiality regulations. All randomized participants will be included in the final primary outcome analysis in accordance with the intention-to-treat (ITT) principle, regardless of protocol deviations, withdrawal, or loss to follow-up, to minimize bias and preserve the randomization benefits. To ensure transparency and reproducibility, the fully de-identified participant dataset, alongside the detailed statistical analysis plan, will be deposited in a publicly accessible repository (e.g., Zenodo or Figshare) upon study completion and manuscript publication.“

To maximize patient retention and minimize dropout rates during the 6-week study cycle, our research coordinators will maintain regular communication with participants via weekly phone calls or WeChat messages. Follow-up assessments will be scheduled flexibly to accommodate patients’ availability, and transportation assistance will be provided if necessary. For data quality control, all clinical assessments will be double-entered into the electronic database by two independent researchers to ensure accuracy. The principal investigator will conduct regular monitoring and logic checks on the case report forms (CRFs) to promptly identify and resolve any missing, inconsistent, or anomalous data.

## Discussion

This study aims to explore the clinical effectiveness, feasibility, and safety of priming iTBS combined with REX exoskeleton rehabilitation robots for restoring lower limb motor function in stroke patients. To our knowledge, it is among the first to systematically investigate this novel combination therapy. The hypothesis that this integrated approach will promote lower limb functional recovery is grounded on the following evidence:

First, the lower-limb representation within the primary motor cortex (M1) is anatomically deep, situated within the longitudinal cerebral fissure. Consequently, localizing this region using standard TMS coils remains methodologically challenging [35]. While traditional figure-of-eight coils struggle to achieve sufficient depth for optimal stimulation, double-cone coils have proven effective in eliciting reliable lower-limb myoelectric responses, despite being underutilized in iTBS protocols. Studies have shown that the double-cone coil is more suitable for the localization and treatment of the lower limb M1 region [36]. Earlier, motor evoked potentials (MEPs) in lower limb muscles were successfully induced using double-cone coils [37,38]; subsequently, Beaulieu, Lewis, Wheaton, et al. further verified the reliability of double-cone coils in lower limb applications among stroke patients [39–41]. Our team’s previous research verified the reliability of TMS double-cone coils in evaluating the lower limb region of the primary motor cortex (M1 region), clarified the minimum number of stimulations required to obtain effective TMS evaluation parameters, and provided evidence for the application of double-cone coils in the lower limbs [42].

Additionally, the combined use of MEP and fNIRS techniques enables a more comprehensive study of the mechanism of action of rTMS. MEP accurately measures changes in excitability in target brain regions (such as the lower limb motor cortex) [43], while fNIRS can monitor changes in whole-brain functional connectivity [44]. These two techniques are complementary: they not only reveal how rTMS drives whole-brain network reorganization after enhancing local excitability, but also, by leveraging the advantage of fNIRS in resisting motion interference, provide better reference indicators for optimizing precise neuromodulation of lower limb motor dysfunction in stroke.

Effective post-stroke motor rehabilitation must overcome the inherent limitations of single-target interventions. Therefore, “central-peripheral-central” (CPC) closed-loop theory [45] and the “brain-limb synergy” theory [46] offer a robust theoretical framework for integrating central neuromodulation with peripheral motor training.

The CPC closed-loop theory emphasizes the dynamic interaction between central interventions (such as transcranial magnetic stimulation) and peripheral interventions (such as functional electrical stimulation and rehabilitation training): central interventions enhance neural plasticity by activating brain regions, while peripheral interventions strengthen central remodeling through sensory-motor signal feedback, forming a closed loop of “activation-feedback-remodeling” [45].

Studies have shown that TBS combined with Neuromuscular Electrical Stimulation (NMES) as central plus peripheral stimulation showed synergistic effect in both electrophysiological and clinical assessment. [47]. This theory reveals that the essence of stroke rehabilitation lies in the bidirectional regulation between the central and peripheral systems, providing a theoretical foundation for multimodal interventions. Moreover, the “brain-limb synergy” theory [46] proposes that the synergistic effect of central and peripheral technologies depends on intervention timing, including synchronous synergy (e.g., concurrent brain stimulation and walking training) and asynchronous synergy (e.g., brain stimulation followed by limb training). To clarify the optimal timing of rTMS and physical therapy, Avenanti et al. [48] randomly assigned 30 chronic stroke patients into 4 groups. These groups received either real or sham rTMS either before (rTMS-PT) or after (PT-rTMS) physical therapy (PT). The results indicated that the rTMS-PT group not only achieved the optimal therapeutic effect but also maintained the effect for the longest duration, and the sequential mode of central intervention followed by peripheral training can enhance the efficiency of motor cortex connectivity. This theory emphasizes that “brain-limb synergy” is not a simple superposition of technologies, but a timing optimization based on the laws of neural plasticity.

In recent years, robotic training has become a cost-effective intervention due to its characteristics of high repeatability, controllable dosage and intensity [49]. Currently, exoskeleton rehabilitation robots have attracted widespread attention in the field of lower limb rehabilitation for stroke survivors [50]. Studies have shown that such training may help improve gait, walking ability, and balance, reduce lower limb muscle spasticity, and enhance cardiopulmonary function in individuals after stroke [51]. A meta-analysis indicated that exoskeleton-assisted gait training is equivalent to or more beneficial than traditional rehabilitation methods in restoring gait and balance in stroke patients [52].

Based on the CPC closed-loop theory, iTBS, which targets the lower limb primary motor cortex (M1) through a double-cone coil, can induce Long-Term Potentiation to enhance cortical excitability, thereby providing a central initiating signal. The REX exoskeleton robot training forms a peripheral feedback loop through proprioceptive input. The signals generated by it are transmitted back to the central nervous system via the thalamocortical pathway, which can optimize the synaptic modifications induced by iTBS, thereby forming an “activation-feedback-remodeling” closed-loop system and achieving bidirectional regulation between the central and peripheral systems. The sequential model of iTBS followed by REX exoskeleton robotics training fits the requirements of the brain-limb synergy theory in terms of the intervention sequence. By priming the central motor cortex into a pre-facilitated state, iTBS enhances the brain’s receptivity to subsequent peripheral inputs. When followed immediately by REX exoskeleton training, this sequential approach allows for the highly efficient integration of peripheral sensory signals. This synergistic interaction strengthens functional connectivity across brain regions, establishing a positive “brain activation - limb output” feedback loop that is critical for rebuilding lower-limb motor function.

A notable limitation is that this study strictly enrolls patients with their first-ever stroke, excluding recurrent cases. Although this ensures sample homogeneity by minimizing confounding from prior brain lesions, it inevitably limits the generalizability of our findings. Future trials should validate this protocol in broader, more heterogeneous stroke populations.

By integrating the dual advantages of central neuromodulation and peripheral motor training, this protocol is expected to provide a more precise and efficient new intervention approach for the rehabilitation of lower limb motor function in stroke patients.

## Supporting information

S1 File SPIRIT Checklist. (DOC)

S2 File Standard Operating Procedure (SOP). (DOCX)

S3 File Clinical Trial Protocol. (DOCX)
