# Supplementary material for: Priming iTBS for lower limb rehabilitation after stroke: Protocol for a randomized controlled trial on efficacy and neuroplasticity
Source: PLoS One. 2026 May 21;21(5):e0349578. doi: 10.1371/journal.pone.0349578 (PMC13193341; doi:10.1371/journal.pone.0349578)
Supplement: S2 File — (DOCX) [file pone.0349578.s002.docx]

**Standard Operating Procedure (SOP)**

[**1. INTERVENTION** 2](#_Toc211961261)

[**1.1 Conventional Treatment** 2](#_Toc211961262)

[**1.1.1. Physical Therapy (PT) Protocol** 2](#_Toc211961263)

[**1.1.2. Occupational Therapy (OT) Protocol** 3](#_Toc211961264)

[**1.2 Robot-assisted Training** 4](#_Toc211961265)

[**1.2.1 Equipment Information** 4](#_Toc211961266)

[**1.2.2 Training Programs (Refined Operational Procedures, Parameters, and Monitoring)** 4](#_Toc211961267)

[**1.3 Transcranial Magnetic Stimulation (TMS) Intervention** 7](#_Toc211961268)

[**1.3.1 Equipment Information** 7](#_Toc211961269)

[**1.3.2 Pre-intervention Preparation** 8](#_Toc211961270)

[**1.3.3 Intervention Protocols for Each Group** 10](#_Toc211961271)

[**2. Quality Control** 12](#_Toc211961272)

[**2.1 Role and Responsibility** 12](#_Toc211961273)

[**2.2 Personnel Training** 13](#_Toc211961274)

[**2.3 Intervention Principles** 14](#_Toc211961275)

[**2.4 Data Collection and Evaluation Procedures** 14](#_Toc211961276)

[**3. Adverse Event Management** 15](#_Toc211961277)

[**3.1 Definition of Adverse Events** 15](#_Toc211961278)

[**3.2 Adverse Event Documentation** 15](#_Toc211961279)

[**3.3 Adverse Event Handling** 15](#_Toc211961280)

[**3.4 Reporting Methods** 16](#_Toc211961281)

[**3.5 Withdrawal** 16](#_Toc211961282)

[**4. Equipment and Material Management** 16](#_Toc211961283)

[**4.1 Equipment Management** 16](#_Toc211961284)

[**4.2 Material Management** 17](#_Toc211961285)

[**5. Participant Compliance Management** 18](#_Toc211961286)

[**5.1 Strategies to Improve Compliance** 18](#_Toc211961287)

[**5.2 Compliance Monitoring and Management** 18](#_Toc211961288)

**1. INTERVENTION**

All three groups will receive conventional treatment and robot-assisted training as the basic intervention. On this basis, different combinations of transcranial magnetic stimulation (TMS) will be implemented according to group allocation.

**1.1 Conventional Treatment**

All participants enrolled in the study will undergo a standardized conventional rehabilitation program in addition to their assigned experimental interventions. This protocol, delivered five days per week, includes a daily 30-minute session of Physical Therapy (PT) and a separate 30-minute session of Occupational Therapy (OT). The interventions are tailored to the individual's functional level but maintain standardized core components as detailed below.

**1.1.1. Physical Therapy (PT) Protocol**

**Frequency and Duration:** 5 days per week, 1 session per day, 30 minutes per session.

**Core Components:**

**Strength Training**

**Activities:** Lower limb closed-chain exercises (e.g., mini-squats, static squat holds, heel raises) and upper limb open-chain exercises (e.g., shoulder flexion/abduction, elbow flexion/extension).

**Parameters:** 2-3 sets of 12-15 repetitions per exercise. 60-second rest between sets.

**Progression:** Increase resistance or difficulty when the patient can comfortably complete 3 sets of 15 repetitions without pain or excessive fatigue.

**Balance Training**

**Activities:** Progress from static balance (unsupported sitting, standing, tandem stance) to dynamic balance (standing weight shifts, reaching tasks in different directions).

**Parameters:** Hold each static posture for 30 seconds, repeat 4-5 times. Perform 8-10 repetitions of dynamic reaching tasks.

**Progression:** Gradually reduce external support (e.g., moving from parallel bars to no support) and narrow the base of support as tolerated.

**Transfer and Gait Training**

**Activities:** Practice of sit-to-stand and stand-pivot transfers; overground walking with emphasis on correcting gait deviations; stepping over obstacles (5-10 cm in height).

**Parameters:** 5-8 repetitions of transfer exercises per set. Cumulative walking time of 5-10 minutes per session, or until moderate fatigue is observed.

**Safety:** Therapist must provide close supervision and use a gait belt at all times during standing and walking activities.

**Spasticity and Range of Motion (ROM) Management**

**Activities:** Passive and active-assisted ROM exercises for all major joints on the affected side; slow, sustained static stretching of spastic muscle groups.

**Parameters:** 10-15 repetitions per joint direction for ROM. Hold stretches for 30 seconds, repeating each 3-4 times.

**Precautions:** All movements must be gentle and pain-free. Stop immediately if pain or increased spasticity is elicited.

**1.1.2. Occupational Therapy (OT) Protocol**

**Frequency and Duration:** 5 days per week, 1 session per day, 30 minutes per session.

**Core Components:**

**Basic Activities of Daily Living (ADL) Training**

**Activities:** Practice of grooming (washing face, brushing teeth), dressing (donning/doffing shirts, pants, socks), and self-feeding using adaptive utensils as needed.

**Parameters:** 10-15 minutes of practice per activity.

**Method:** Utilize task breakdown strategies. Therapists provide the minimal assistance necessary to encourage maximum patient independence.

**Upper Limb Function Training**

**Activities:** Task-oriented training (e.g., stacking cups, manipulating objects) and fine motor training (e.g., picking up small items, rotating nuts and bolts).

**Parameters:** 8-10 minutes of practice per task.

**Progression:** Select objects of varying size, weight, and manipulation demand to match and challenge the patient's ability.

**1.2 Robot-assisted Training**

**1.2.1 Equipment Information**

**Device Model:** REX Self-Balancing Exoskeleton Rehabilitation Robot (Rex Bionics Ltd., New Zealand).

**Key Features:** Wearable, self-stabilizing dynamic exoskeleton. Provides support, balance assistance, and adjustable assistance levels based on patient capability.

**Usage Environment:** Conducted in a dedicated, flat, obstacle-free rehabilitation training room.

**Safety Facilities:** The device is equipped with an emergency stop button. Therapists provide continuous supervision throughout the session.

**1.2.2 Training Programs (Refined Operational Procedures, Parameters, and Monitoring)**

**Pre-Training Preparation:**

**Patient Assessment:** Before each session, the physical therapist performs a rapid assessment of the patient's current mental state, fatigue level (using Borg CR10 Scale and VAS 0-10), pain status (VAS 0-10), blood pressure, heart rate, and muscle tone of the affected limb (Modified Ashworth Scale, MAS).

**Equipment Preparation:**

Ensure the REX robot battery level is sufficient, joint range of motion is normal, and the emergency stop function is operational.

Adjust the exoskeleton backplate length, thigh and calf brace lengths, and foot strap tightness according to the patient's height and body build. Ensure comfortable wear without pressure points, and approximate alignment of joint hinges with the patient's anatomical joints.

Assist the patient in donning the robot and securely fasten all straps and safety locks.

**Initial Settings:** Set the initial support level, gait speed, and training difficulty level on the REX control panel based on the patient's current functional level and daily condition.

**Standardized Training Programs and Parameters:**

**Program 1: Standing Activity Training**

**Goal:** To improve trunk control and coordination during stable standing.

**Procedure:**

Patient stands stably wearing the REX robot.

Place target objects (e.g., Bobath balls of different colors, lightweight blocks) at various locations (front, left-front, right-front, left, right) and heights (shoulder height, waist height, knee height) in front of the patient.

Instruct the patient to push the Bobath ball or grasp and place the target objects at designated spots.

Based on patient performance, the therapist progressively increases target distance, alters placement speed (e.g., therapist moves the target), or instructs the patient to perform slight trunk rotation/lateral flexion while executing tasks.

**Parameters:** Each training session lasts 10 minutes. The therapist records in real-time the patient's movement fluidity, trunk sway, and need for additional assistance or robot parameter adjustments.

**Program 2: Resistance Training with Elastic Bands**

**Goal:** Utilize the upper limb PNF diagonal spiral pattern to facilitate extension of the affected lower limb and trunk, enhancing lower limb muscle strength and movement coordination.

**Procedure:**

Patient stands stably wearing the REX robot.

Secure one end of an appropriately resistant elastic band to the unaffected lower limb (e.g., distal thigh). The patient grasps the other end with the unaffected upper limb.

The therapist guides the patient to perform resisted movements with the unaffected upper limb using a specific PNF diagonal pattern (e.g., D2 flexion or extension). Emphasis is placed on utilizing force from the unaffected upper limb to drive trunk rotation and core engagement, thereby promoting weight-bearing and extension of the affected lower limb.

The therapist closely monitors the extension response, weight-bearing status of the affected lower limb, and presence of any synergistic movement patterns.

**Parameters:** Each training session lasts 10 minutes. Select elastic band resistance (color/poundage) based on patient muscle strength. Record the resistance level used and number of repetitions. The therapist observes and records the active participation level of the affected lower limb.

**Program 3: Lower Limb Functional Training**

**Goal:** Enhance lower limb strength, balance ability, and functional mobility.

**Procedure (Select 1-2 items based on patient capability, total duration 10 minutes):**

**Single-leg Weight-bearing:** With REX support, guide the patient to slowly shift weight onto the affected leg and attempt to lift the unaffected leg off the ground, holding for several seconds. Gradually increase hold time and decrease robot support level. Ensure safety.

**Lateral Stepping Training:** Guide the patient to perform small-range lateral steps towards the affected and unaffected sides. Emphasize weight shifting and control. Ground markers can indicate step length.

**Squat Training:** With necessary REX support, guide the patient to perform controlled, small-range knee flexion squats (maintaining upright trunk, knees not exceeding toes), then stand up. Gradually increase squat depth and decrease support level.

**Parameters:** Set specific repetitions for each sub-item (e.g., Single-leg Weight-bearing: 5-10 reps/set on affected side * 2-3 sets; Lateral Stepping: 5-10 steps/set per side * 2-3 sets; Squats: 8-12 reps/set * 2-3 sets). Record completed sets, repetitions, robot support level used, and patient performance (e.g., stability, fatigue).

**In-Session Monitoring and Adjustment:**

**Vital Signs:** Regularly (at least every 10 minutes) inquire about patient sensations and monitor heart rate and respiration. Have a blood pressure monitor and pulse oximeter readily available near the training area.

**Fatigue and Pain:** Assess patient subjective fatigue and any pain using the Borg CR10 Scale or VAS (0-10) in real-time. If fatigue score ≥7, pain score ≥4, or signs like pallor, rapid breathing, or profuse sweating occur, immediately pause training for rest and assessment.

**Difficulty Adjustment:** Based on real-time patient performance and feedback, the therapist dynamically adjusts the support level, stability assistance level, or training speed on the REX control panel. The goal is to maintain the patient in a "challenging but achievable" state.

**Feedback:** The therapist provides continuous, specific, and positive verbal feedback (e.g., "Shift your weight a bit more to the left," "Good knee extension!") and guides the patient to focus on correct movement sensations.

**Post-Training Procedures:**

Assist the patient in slowly and safely doffing the robot.

Record specific parameters for each program during the session (support settings, completion status, patient response, adjustments made), patient fatigue level (Borg/VAS), and any discomfort.

Briefly inform the patient about training outcomes and the plan for the next session.

**Overall Parameters:** The three programs are performed sequentially. Each program lasts 10 minutes, totaling 30 minutes per day. Sessions occur 5 days per week (Monday to Friday) for 4 weeks. Executed and recorded by experienced physical therapists who have completed specific project training.

**1.3 Transcranial Magnetic Stimulation (TMS) Intervention**

**1.3.1 Equipment Information**

**Device Model:** Wuhan Yiruidian Paired Transcranial Magnetic Stimulator, Model: N50000.

**Coil:** Matched double-cone coil.

**Navigation System:** Equipped with an electromagnetic positioning system for precise recording and reproduction of the stimulation target.

**1.3.2 Pre-intervention Preparation**

**Preparation:**

**Environment & Safety:** Conducted in a quiet, dedicated TMS room with minimal electromagnetic interference. Remove all metal objects from the patient (including hearing aids, credit cards). Have an emergency kit (containing anti-seizure medication e.g., diazepam), oxygen supply, and suction apparatus readily available. Therapists must be familiar with emergency procedures.

**Patient Verification & Consent Review:** Re-confirm patient identity, group allocation, and absence of TMS contraindications (history of epilepsy, intracranial metal implants, cardiac pacemakers, etc.). Briefly reiterate potential discomforts (noise, scalp sensation).

**Equipment Check:** Power on and preheat the device. Verify the cooling system is functioning. Connect and calibrate the navigation system.

**Target Localization ("Hotspot" Localization):**

**Target Muscle:** Affected Tibialis Anterior (TA) muscle.

**Initial Positioning:** Patient positioned comfortably seated or semi-reclined, affected lower limb relaxed.

**Anatomical Landmarks:** Initially target an area approximately 2-3cm posterior and 1-2cm lateral to the "Cz" point (International 10-20 EEG system) on the affected hemisphere.

**EMG Setup:** Clean skin. Place surface EMG electrodes (DELSYS) on the belly of the affected TA muscle (recording electrode) and the Achilles tendon (reference electrode). Ensure impedance <5kΩ. Set EMG amplifier parameters (e.g., Gain 1000x, Bandwidth 20-2000 Hz). Ensure clear display of raw EMG and MEP signals on the TMS device software.

**Localization Procedure:**

1. Set stimulator output intensity to approximately 50-60% of Maximum Stimulator Output (MSO).
2. Apply single-pulse TMS stimulation within the initial target area and surrounding (±1cm grid).
3. Observe and record elicited MEPs (Motor Evoked Potentials). The goal is to elicit consistent MEPs with peak-to-peak amplitude ≥50 μV in at least 5 out of consecutive stimuli.
4. "Hotspot" Determination: Identify the precise scalp location eliciting the largest and most stable MEP amplitude. Record the 3D coordinates of this point (relative to anatomical landmarks like Cz/Nz) using the navigation system and save it.

**Motor Threshold Determination:**

**Resting Motor Threshold (rMT):**

**State:** Affected TA muscle completely relaxed (confirmed by real-time EMG monitoring showing electrical silence).

**Definition:** The minimum stimulus intensity (%MSO) required to elicit MEPs with a peak-to-peak amplitude ≥50 μV in at least 5 out of 10 consecutive stimuli.

**Determination Method (Staircase Procedure):**

1. Starting Intensity: 50% of estimated threshold or 30% MSO (if no reference value).
2. Increase or decrease intensity in 1% MSO steps.
3. Near threshold, deliver 4-6 stimuli per intensity level (not necessarily 10 consecutive for efficiency).
4. Record the lowest intensity that consistently elicits MEPs ≥50 μV as the rMT.

**Active Motor Threshold (aMT):**

**State:** Patient maintains a slight voluntary contraction of the affected TA muscle (~20% of Maximum Voluntary Contraction, MVC), aided by visual EMG biofeedback.

**Definition:** The minimum stimulus intensity (%MSO) required to elicit MEPs with a peak-to-peak amplitude ≥200 μV in at least 5 out of 10 consecutive stimuli during voluntary contraction.

**Determination Method:** Same as rMT staircase procedure.

**Recording:** Accurately record the measured rMT and aMT values (%MSO), noting the date of determination. For multiple interventions, the need for re-testing thresholds should be explicitly defined in the protocol (usually once at baseline, re-tested if threshold change is suspected or after long intervals).

**1.3.3 Intervention Protocols for Each Group**

**General Rules:**

**Sequence:** All TMS interventions are performed immediately before each robot-assisted training session. The interval between them should be minimized (<15 minutes).

**Coil Placement:** Use the navigation system to ensure coil position and angle (tangential to the scalp) are identical to the "hotspot" localization.

**Stimulus Intensity:** Set based on the determined aMT (see group descriptions below).

**Patient State:** During stimulation, patients remain quiet, awake, and relaxed (unless the protocol requires active contraction). Monitor EMG continuously (especially during iTBS).

**Recording:** After each TMS session, record actual stimulus parameters used (intensity, frequency, number of pulses, duration), coil position (coordinates or markers), patient tolerance, and any adverse events.

**Specific Protocols per Group:**

**Group 1: Priming iTBS Group (cTBS+iTBS)**

**Step 1: cTBS (Continuous Theta Burst Stimulation - Inhibitory)**

**Pattern:** 50 Hz triplet pulses repeated at 5 Hz.

**Parameters:** 200 uninterrupted bursts, Total Pulses: 600.

**Duration:** ~40 seconds.

**Stimulus Intensity:** **80% aMT**.

**Target Brain Area:** "Hotspot" in the lower limb M1 area of the **ipsilesional hemisphere**.

**Step 2: iTBS (Intermittent Theta Burst Stimulation - Excitatory)**

**Pattern:** 2-second burst (3 pulses at 50 Hz) followed by 8-second rest (one 10-second cycle).

**Parameters:** **20 cycles**, **Total Pulses: 600** (20 cycles * 30 pulses/cycle).

**Duration:** ~192 seconds (20 cycles * 10 seconds/cycle).

**Stimulus Intensity:** **80% aMT**.

**Target Brain Area:** "Hotspot" in the lower limb M1 area of the **ipsilesional hemisphere**.

**Interval:** iTBS begins immediately after cTBS ends.

**Group 2: Non-Priming iTBS Combined Group (sham cTBS + iTBS)**

**Step 1: Sham cTBS**

**Key:** Mimics the sound and partial somatosensation of real cTBS, no effective magnetic field.

**Procedure:**

1. Position the TMS coil perpendicular to the scalp surface (ensured using the navigation system's distance lock or a physical spacer).
2. Load the sham cTBS parameters onto the device. The stimulator's interface will display identical parameters to the active condition (200 bursts, 600 pulses, ~40 sec duration, intensity "displayed" as 80% aMT); however, the device was preset to sham mode, ensuring that no actual magnetic stimulation was delivered despite the visual feedback. The device emits the same sound as real stimulation (coil discharge click).
3. The patient may feel a faint scalp vibration (caused by coil vibration), but there is no intracranial induced electric field or neuromodulatory effect.

**Target Brain Area:** Nominally targets the "hotspot" in the lower limb M1 area of the **ipsilesional hemisphere**, but effectively inert.

**Step 2: iTBS (Same as Group 1 Step 2)**

**Real Stimulation**

**Parameters:** Same as Group 1 Step 2 (20 cycles, 600 pulses, ~192 sec, 80% aMT).

**Target Brain Area:** "Hotspot" in the lower limb M1 area of the **ipsilesional hemisphere**.

**Interval:** iTBS begins immediately after sham cTBS ends.

**Group 3: Sham Stimulation Group (sham cTBS + sham iTBS)**

**Step 1: Sham cTBS**

Procedure same as Group 2 Step 1.

**Target Brain Area:** Nominally targets the **ipsilesional hemisphere**.

**Step 2: Sham iTBS**

**Procedure:**

1. Position the TMS coil perpendicular to the scalp surface (ensured using the navigation system's distance lock or a physical spacer).
2. Load the sham iTBS parameters onto the device. The stimulator's interface will display identical parameters to the active condition (20 cycles, 600 pulses, ~192 sec duration, intensity "displayed" as 80% aMT); however, the device was preset to sham mode, ensuring that no actual magnetic stimulation was delivered despite the visual feedback. The device emits the same sound as real iTBS stimulation.
3. The patient feels a faint scalp vibration, no effective neuromodulatory effect.

**Target Brain Area:** Nominally targets the "hotspot" in the lower limb M1 area of the ipsilesional hemisphere, but effectively inert.

**Interval:** Sham iTBS begins immediately after sham cTBS ends.

**2. Quality Control**

**2.1 Role and Responsibility**

**Principal Investigator (PI)**: Responsible for reviewing the consistency between intervention procedures and data; oversees ethical compliance and handles safety-related events.

**Clinical Medical Coordinator (CMC)**: Responsible for recruiting participants, conducting baseline assessments, and ensuring informed consent is obtained.

**Clinical Research Coordinator:** Responsible for accessing the central web-based randomization system after a participant's baseline assessment is completed. The system will then automatically reveal the group allocation only to the unblinded physical therapist responsible for administering the intervention, ensuring allocation concealment.

**Statistician (for randomization)**: Responsible for implementing the randomization procedure using a central randomization system.

**Physical Therapists**: Responsible for delivering robot-assisted training and TMS interventions, adjusting training difficulty, and providing feedback.

**Safety Supervisor**: Monitors safety of intervention procedures; responsible for managing adverse events.

**Rehabilitation Assessor**: Performs scheduled assessments (at T0, T1, T2, T3) of enrolled participants, remains blinded to group allocation, and conducts assessments independently.

**Data Monitoring Committee (DMC)**: Responsible for overseeing the study process, regularly reviewing data, and ensuring data quality.

**Data Entry Personnel**: Responsible for data entry into case report forms (CRF) and electronic database, ensuring data accuracy and confidentiality.

**Outcome Statistician**: Conducts statistical analysis of the trial outcomes using SPSS 22.0 software.

**2.2 Personnel Training**

All research personnel involved in the trial must undergo training in Good Clinical Practice (GCP), covering ethical principles of clinical research, regulatory requirements, and data management standards. The training duration shall be no less than 8 hours, followed by a written examination. Only those who pass the assessment will be permitted to participate in the trial.

**Clinical Medical Coordinator (CMC)**: Must hold a valid clinical physician licence and have more than three years of clinical experience. Training includes participant recruitment, scale-based assessments (such as FMA-LE, BBS, PASS, MBI), and documentation.

**Physical Therapists**: Must hold a valid rehabilitation therapist certification with at least three years of clinical experience. Training covers robot-assisted training procedures, TMS operation (including hotspot localization, motor threshold determination, and stimulation parameter setting), and adverse event identification. Training is delivered through lectures, video demonstrations, and simulation-based practice, with a minimum duration of 3 hours. Personnel must pass a simulation assessment before participating.

**Safety Supervisor**: Must hold a valid clinical physician licence and have at least three years of clinical experience. Training focuses on the identification and management of adverse events related to TMS and robot-assisted training.

**Rehabilitation Assessor**: Must hold a valid qualification as a rehabilitation or clinical physician, with at least 5 years of experience in rehabilitation practice. Must complete professional training and pass an assessment on the administration and scoring of outcome measures, including FMA-LE, BBS, PASS, MBI, and MEP-related parameters. Assessors must remain blinded to group allocation.

**Data Manager**: Should have a background in medicine, nursing, or a related discipline and be familiar with clinical trial data collection procedures. Responsible for database creation, data entry, coding, and confidentiality management.

**2.3 Intervention Principles**

All therapists delivering the interventions must complete standardized training, which includes at least 3 simulation sessions and 1 competency assessment per person. TMS stimulation parameters (intensity, frequency, pulse number, and duration) and robot-assisted training programs must be strictly implemented according to the standard operation card.

The DMC conducts biweekly spot checks on physical therapists’ TMS procedures and robot-assisted training operations to verify adherence to the SOPs. These spot checks involve onsite observations, covering no less than 20% of treatment cases each time. Any procedural deviations identified will be corrected promptly, and retraining will be provided as necessary.

**2.4 Data Collection and Evaluation Procedures**

Outcome assessments are conducted at baseline (T0), midway at 2 weeks (T1), immediately post-intervention at 4 weeks (T2), and follow-up at 6 weeks (T3) by two fixed rehabilitation assessors alternating duties to minimise bias.

**Assessment tools**: FMA-LE, PASS, MBI, fNIRS, three-dimensional gait analysis, sEMG, EEG and MEP.

**Data entry and management**: All evaluation data are initially entered into case report forms (CRF) and synchronized to the electronic database. Double entry by two personnel is required for key data, with a third person performing random audits. Each participant is assigned a unique research identifier, and all data are stored in a de-identified manner.

**Data review**: The DMC regularly reviews the data according to the monitoring plan and written SOPs, verifying consistency, logic, and validity of data entry and storage. Data entered are cross-checked against source documents, and any discrepancies are promptly investigated and rectified.

**Confidentiality**: During data utilisation and analysis, participant personal information is anonymised by removing identifying details such as name, ID number, and contact information, retaining only key data required for research.

**3. Adverse Event Management**

**3.1 Definition of Adverse Events**

An adverse event refers to any unintended physical or psychological discomfort experienced during the study, regardless of its causal relationship with the intervention, including but not limited to hearing problems, local pain, muscle twitching, joint soreness, muscle fatigue, dizziness, and seizures.

**3.2 Adverse Event Documentation**

After each treatment session, patients’ conditions will be closely monitored. Any adverse events, including their onset time, manifestations, relevant circumstances (such as intervention being performed, intensity, etc.), and outcomes, will be promptly and accurately recorded, with an analysis of potential causes.

**3.3 Adverse Event Handling**

**Seizures**: This is the most severe acute adverse effect of iTBS. If a seizure occurs, immediately stop the intervention, ensure the patient's airway is unobstructed, protect the patient from injury, and call for emergency medical assistance. Monitor vital signs and provide necessary first aid.

**Hearing problems**: If the patient reports hearing discomfort or tinnitus, stop the intervention, check the device's sound output, and have the patient rest in a quiet environment. If symptoms persist, refer to an otolaryngologist.

**Local pain, muscle twitching, joint soreness, muscle fatigue**: Reduce the intensity or duration of the intervention, allow the patient to rest, and apply appropriate physical therapy (such as cold or heat therapy) to relieve symptoms. Adjust the intervention parameters in subsequent sessions based on the patient's tolerance.

**Dizziness**: Stop the intervention, assist the patient to lie down or sit comfortably, monitor blood pressure and heart rate. If dizziness persists or is severe, conduct further medical examinations to rule out other causes.

**3.4 Reporting Methods**

Severe adverse events (such as seizures) must be reported promptly to the ethics committee of Yancheng First People's Hospital and other relevant authorities in strict accordance with ethical guidelines within 24 hours of occurrence.

**3.5 Withdrawal**

In cases of severe adverse events, withdrawal from the study may be decided by the DMC after an SOP meeting. Unblinding procedures will be performed for participants who withdraw. For participants who withdraw, attempt to collect the final medical assessment data and basic information to ensure data completeness, and inform them that they will continue to receive necessary medical advice and support.

**4. Equipment and Material Management**

**4.1 Equipment Management**

**Equipment inventory**: Clearly list all equipment required for the trial, including the Wuhan Iridium paired transcranial magnetic therapy device (model: N50000) and matching conical coil, REX self-balancing exoskeleton rehabilitation robot, functional near-infrared spectroscopy device (NirSmart-3000B), functional electrical stimulation workstation (model YSA03P/02P, brand: YASI), etc.

**Calibration and maintenance**: Establish a regular calibration schedule. The transcranial magnetic therapy device is calibrated every 6 months by the manufacturer’s technical staff, verifying accuracy of output frequency, intensity, and other parameters. The robot-assisted training device and functional electrical stimulation workstation undergo annual calibration. A maintenance logbook is maintained to record device inspections before and after each use, including operational status and usage duration. Any equipment malfunction must be promptly reported to maintenance personnel for repair, with detailed records of the fault, repair time, and parts replaced.

**Performance verification**: Before each use of the transcranial magnetic therapy device, functional near-infrared spectroscopy device, and functional electrical stimulation workstation, perform performance verification using standard methods to confirm accurate signal acquisition and recording. If verification fails, the device must not be used until recalibrated or repaired.

**4.2 Material Management**

**Material inventory**: List all materials required for the trial, such as surface EMG electrodes (DELSYS), disposable gloves, alcohol swabs, etc.

**Procurement and acceptance**: Define quality standards for materials procurement. Surface EMG electrodes must have good conductivity and no allergic reactions. Upon arrival, materials undergo acceptance by designated personnel, checking specifications, quantity, intact packaging, and verifying product certificates and quality inspection reports. Only accepted materials are stored; non-conforming materials are returned to the supplier.

**Storage and usage**: Materials should be stored in a dry, ventilated environment. Disposable items (such as gloves, alcohol swabs) should be kept in sterile containers and replaced regularly.

**Expiry management**: Maintain a ledger of material expiry dates and conduct regular checks. Materials approaching expiry should trigger advance warnings. Expired materials are prohibited from use and must be disposed of according to medical waste regulations, with disposal records maintained.

**Usage records**: Each time materials are used, verify inventory and expiry dates to ensure proper utilisation.

**5. Participant Compliance Management**

**5.1 Strategies to Improve Compliance**

**Health Education**: Provide diversified and personalised health education guidance for patients and their caregivers, including the study process, intervention methods, expected benefits, and importance of adherence. Utilise multimedia approaches such as animated videos and audio lessons, combined with printed manuals and illustrated posters. Explain the relationship between compliance and rehabilitation effects using simple language and case examples.

**Psychological Support**: Establish a regular communication mechanism with patients and their families to understand their psychological state. Arrange regular counselling sessions by healthcare staff to address concerns and doubts, share rehabilitation success stories, and enhance patients’ confidence in recovery. Offer emotion regulation techniques (e.g., deep breathing exercises) to help patients cope with stress during the intervention.

**5.2 Compliance Monitoring and Management**

**Treatment Record Monitoring**: Keep detailed records of each participant’s attendance, duration, content, intensity, and completion status of TMS interventions and robot-assisted training. Calculate quantitative indicators from treatment records:

Treatment completion rate = (Actual number of completed treatments / Planned number of treatments) × 100%

Assessment participation rate = (Actual number of completed assessments / Planned number of assessments) × 100%

Follow-up Retention Rate = (Number of on-time follow-ups / Planned number of follow-ups) × 100%

**Management of Non-compliance**: For participants exhibiting non-compliance, initiate communication within 48 hours via face-to-face interview, phone call, or video consultation to explore reasons such as fatigue, pain, psychological stress, or cognitive impairment, and implement targeted measures. Reiterate the significance and potential benefits of the intervention, tailored to the patient’s situation. Convene family meetings to develop home support plans, clarifying family members’ responsibilities in supervising the patient's adherence to the intervention schedule and providing emotional support. For severe non-compliance cases where continuation is not possible, respect patient autonomy while attempting to collect relevant data and follow the study protocol for participant withdrawal procedures.
