# Supplementary material for: Priming iTBS for lower limb rehabilitation after stroke: Protocol for a randomized controlled trial on efficacy and neuroplasticity
Source: PLoS One. 2026 May 21;21(5):e0349578. doi: 10.1371/journal.pone.0349578 (PMC13193341; doi:10.1371/journal.pone.0349578)
Supplement: S3 File — (DOCX) [file pone.0349578.s003.docx]

**Research Protocol**

(Version Number: V1.0 Version Date: August 1, 2025)

**Project Name:**

Efficacy of iTBS on lower limb motor dysfunction in stroke patients after pre-modulation and the mechanism of brain network remodelling

**Sponsor Unit:** School of Rehabilitation Medicine, Jiangsu Medical College

**Responsible Department:** Department of Rehabilitation, Yancheng No.1 People's Hospital

**Principal Investigators:** Bin Su; Ya Zhou

**Research Duration:** September 2025 – September 2028

**1. Study Subjects**

Recruit 60 stroke patients hospitalized in the Department of Rehabilitation Medicine, Yancheng First People's Hospital.

**1.1 Inclusion Criteria**

(1) Stroke diagnosed by CT or MRI.

(2) Age 40-80 years old.

(3) Stable condition, first onset of illness, duration 1-3 months.

(4) Able to walk independently or with assistance for more than 10m, Brunnstrom stage III-V lower limb.

(5) Cognitive clarity, ability to follow simple verbal commands or instructions, MoCA≥26.

(6) Informed consent signed by the patient or his/her relatives.

(7) No history of epilepsy.

(8) No contraindications to TMS examination.

**1.2 Exclusion Criteria**

(1) Individuals with relevant contraindications to TMS (pacemakers, intracranial implants, implanted drug pumps etc.).

(2) Significant speech, attention, auditory, visual (lateral neglect, hemianopsia, etc.), intellectual, psychiatric or cognitive dysfunction.

(3) Prior history of organic brain disease, neuropsychiatric history, drug abuse and alcoholism. (4) Patients with severe failure of vital organs (e.g., heart, lung, liver, kidney).

(5) Patients with pre-existing lower limb motor dysfunction prior to the stroke.

(6) Those who are currently participating in other clinical trials.

(7) Those who cannot co-operate with the functional assessment and have poor compliance. (8) Inability to induce a TMS response in the tibialis anterior muscle prior to formal testing.

(9) Pregnancy.

(10) Use of drugs that affect cortical excitability (e.g., benzodiazepines, baclofen, antiepileptics, certain antidepressants) that cannot be safely tapered or washed out prior to study participation.

**2. Sample Size Estimation**

Assuming the endpoint FMA-LE scale value in the preconditioned iTBS group is 3 points higher than in the non-preconditioned iTBS group, and the combined standard deviation for all groups is 2.5 points. Based on existing research results and clinical experience, at a statistical significance level of α=0.05, to achieve 90% statistical power, and considering a 20% follow-up loss rate, calculation using PASS 15 software indicates: at least 20 patients are needed per group, totaling 60 patients.

**3. Random Grouping**

A total of 60 eligible stroke patients will be enrolled in this study. Following the baseline assessment, a computer-generated randomization sequence will be used to assign all participants in a 1:1:1 ratio to one of three parallel intervention groups: the priming iTBS group (n=20), the non-priming iTBS group (n=20), or the sham stimulation group (n=20). The allocation sequence will be concealed from the researchers enrolling participants. After a participant provides informed consent and all baseline assessments (T0) are completed, the research coordinator will access the central web-based system. The system will then reveal the group allocation only to the unblinded physical therapist responsible for administering the intervention. This ensures that outcome assessors and participants remain blinded.

**4. Intervention Methods**

The intervention will be administered five times weekly over a four-week period.

**4.1 iTBS Intervention Protocol**

Before each robot-assisted training session begins, TBS intervention will be administered using the Wuhan Yiruide paired transcranial magnetic therapy instrument (Model: N50000) and its matching double-cone coil. Stimulation will target the "hotspot" within the lower limb motor cortex M1 area. This location will be determined by identifying the position that produces the maximum motor evoked potential (MEP) amplitude in the tibialis anterior muscle, supplemented by resting motor threshold (rMT) testing.

The intervention protocol will consist of two consecutive segments: priming (using cTBS) followed by activation (iTBS). The cTBS phase will deliver 600 pulses in approximately 40 seconds, using 200 sets of continuous stimulus trains, with a pulse frequency of 50Hz and a repetition frequency of 5Hz. The iTBS phase will then be implemented: sending 2-second pulse trains of 3 pulses at 50Hz frequency, followed by an 8-second rest period. This 10-second cycle is repeated 20 times, totaling 600 pulses sent in approximately 192 seconds. All stimulation intensities are set to 80% of the individual active motor threshold (aMT). This threshold is defined as the minimum intensity required to elicit MEPs with peak-to-peak amplitude exceeding 200μV in the tibialis anterior muscle in at least 5 out of 10 trials.

For sham stimulation, the same double-cone coil as used for active stimulation will be employed. This coil will be configured for special sham stimulation mode, simulating the audible "click" sound produced by active TMS without delivering a clinically significant magnetic field to the scalp. The coil will be placed in the exact same scalp position and at the same angle as during active stimulation, using the same fixation bracket to ensure consistent positioning and implementation of blinding.

**4.2 Robot-Assisted Training Intervention Protocol**

All patients will receive a standardized robot-assisted training program using the REX self-balancing exoskeleton rehabilitation robot (Rex Bionics Ltd., New Zealand). All robot-assisted training will be conducted after the TBS intervention, and the training interval should be as short as possible (less than 15 minutes). The daily 30-minute training consists of three separate 10-minute programs:

Program 1: Standing Activity Training: This session focuses on improving trunk control ability and upper-lower limb coordination during stable standing, trained through tasks such as targeted reaching.

Program 2: Elastic Band Resistance Training: Utilizing Proprioceptive Neuromuscular Facilitation (PNF) patterns, aiming to promote extension function of the affected lower limb, enhancing muscle strength and coordination.

Program 3: Lower Limb Functional Training: Through exercises such as single-leg weight bearing, lateral stepping, and deep squats, to improve functional strength, balance ability, and mobility.

All robot-assisted training is supervised throughout by experienced physical therapists. The therapist dynamically adjusts the robot assistance intensity and training difficulty based on the patient's real-time performance and tolerance, while providing immediate verbal feedback.

**5. Evaluation Indicators**

Assessments are conducted at the following time points: pre-intervention (T0), post-intervention at 2 weeks (T1), 4 weeks (T2), and 6-week follow-up (T3). The assessors are not involved in the research process. These assessors have over 5 years of clinical experience and are proficient in the application of assessment tools and methods. Assessors are blinded to group assignment.

**5.1 Primary Outcome Measure**

Fugl-Meyer Assessment for Lower Extremity (FMA-LE): Used to quantitatively assess the impairment and recovery of lower limb motor function in stroke patients, systematically evaluating dimensions such as reflexes, synergistic movements, and coordination. This scale uses a 3-level ordinal score (0=severe dysfunction, 1=partial dysfunction, 2=no dysfunction), with a total score of 34 points. It has good reliability and validity and is a widely recognized clinical tool for assessing lower limb motor function.

**5.2 Secondary Outcome Measures**

(1) Postural Assessment Scale for Stroke (PASS): A postural control assessment tool specifically designed for stroke patients, containing 12 items covering static posture maintenance, dynamic center of gravity transfer, body position changes, and anti-interference ability. Each item is scored from 0-3 based on the patient's ability to complete it, with a higher total score indicating better postural control ability.

(2) Modified Barthel Index (MBI): The Barthel Index assesses patients' activities of daily living, including feeding, bathing, grooming, dressing, bowel and bladder control, toileting, bed-chair transfer, walking on level ground, and ascending/descending stairs. The total score is 100 points, with a higher score indicating better ability in activities of daily living.

**5.3 Neurophysiological and Biomechanical Indicators**

(1) Functional Near-Infrared Spectroscopy (fNIRS):

- Device: NirSmart-3000B fNIRS system (Danyang Huichuang Medical Equipment Co., Ltd.)
- Parameters: 16 light sources, 18 detectors (40 effective channels total), dual wavelength (730nm & 850nm), sampling frequency 11Hz
- Procedure: Before data acquisition, patients rest in a quiet, relaxed state for about 1 minute to ensure hemodynamic baseline stability, followed by an 8-minute resting-state fNIRS scan, during which they remain awake and still.
- Analysis Indicators: Primarily analyze changes in oxygenated hemoglobin and deoxygenated hemoglobin concentrations in various brain regions, and assess the functional integration of motor-related cortical networks through the amplitude of low-frequency fluctuations and functional connectivity analysis based on HbO₂ signals.

(2) Electroencephalography (EEG):

- Device: GES 400 high-density 64-channel EEG system (EGI, USA)
- Parameters: Electrodes arranged according to the International 10-20 system, referenced to Cz, sampling rate ≥8 kHz/channel, input noise ≤1μV
- Procedure: Before acquisition, patients rest quietly for 1 minute, followed by a 5-minute closed-eye resting-state EEG recording.
- Analysis Indicators: After signal preprocessing using EEGLAB toolbox, focus on the absolute power values in the alpha frequency band for brain regions related to motor function (electrode sites C3, C4, FC1, FC2, FCz, FC3, FC4).

(3) Three-Dimensional Gait Analysis:

- Device: Qualisys three-dimensional motion capture system
- Technology: Built-in 3D motion sensor adaptive technology, automatically tracking step frequency and gait through computer learning algorithms, precisely detecting multi-dimensional angle changes.
- Analysis Indicators: Automatically collect and analyze basic gait parameters (step frequency, step time, stride length, etc.), and record usage data after a single startup.

(4) Surface Electromyography (sEMG):

- Device: DELSYS Trigno™ wireless biofeedback system (USA)
- Tested Muscles: Rectus femoris, biceps femoris, tibialis anterior, gastrocnemius.
- Analysis Indicators: Root Mean Square value (reflects the overall level of muscle electrical activity in a specific period, related to motor unit recruitment and synchronization of muscle fiber discharge); Median Frequency (the frequency value that divides the EMG signal power spectrum area in half. It reflects the median level of muscle fiber discharge rate, commonly used to assess local muscle fatigue and the mobilization of different types of muscle fibers).

(5) Motor Evoked Potentials (MEP):

- Device: Wuhan Yiruide Transcranial Magnetic Therapy Instrument (N50000) and matching double-cone coil (VCZ001)
- Electrode Placement: According to SENIAM guidelines, recording electrode placed on the belly of the tibialis anterior muscle, reference electrode on the medial malleolus, ground electrode on the contralateral limb.
- Measurement Parameters:
  - Resting Motor Threshold (rMT): The minimum stimulation intensity required to elicit MEPs with peak amplitude ≥50 μV in the relaxed TA muscle in at least 5 out of 10 consecutive stimulations.
  - Active Motor Threshold (aMT): The minimum stimulation intensity required to elicit MEPs with peak amplitude ≥200 μV in the slightly voluntarily contracted TA muscle (about 20% maximum voluntary contraction) in at least 5 out of 10 consecutive stimulations.
  - Basic MEP Parameters: Obtain at least 15 valid MEPs at 130% rMT intensity, record amplitude and latency.
  - Recruitment Curve: Stimulation intensity increases from 80% rMT to 150% rMT, 5 stimuli given at each 10% intensity level, analyze parameters such as RC slope.

**6. Statistical Analysis**

SPSS 22.0 software will be used. Demographic and baseline characteristics will be compared using one-way analysis of variance (ANOVA) or Fisher's exact test. Mixed-design ANOVA (between-subjects factor: group; within-subjects factor: time) and the interaction between time and group will be analyzed to compare dependent outcomes across groups at the three time points (T0-T0, T1-T0, and T2-T0). The significance level is set at P < 0.05. If any significant time-by-group interaction effects are found, pairwise t-tests will be used for comparisons, comparing baseline changes with a Bonferroni correction threshold of 0.017. If any significant time-by-group interaction effects are found, exploratory subgroup analyses will be conducted to investigate potential differential effects among different functional groups. Due to the exploratory nature of the study, we applied the Bonferroni correction only in the post-hoc comparisons of the primary outcome (P=0.017, number of comparisons=3).

**7. Safety Monitoring and Adverse Event Handling**

**7.1 Safety Monitoring**

**7.1.1 Monitoring Entities and Responsibilities**

- Safety Supervisor: Must hold a valid clinical physician license and have ≥3 years of clinical experience, responsible for monitoring the safety of the intervention process, focusing on risks related to TMS and robot-assisted training, and leading adverse event management.
- Physical Therapist: When performing TMS interventions and robot-assisted training, assess the patient's mental state, fatigue, pain, blood pressure, heart rate, and muscle tone in the affected limb before intervention using scales such as Borg CR10, VAS, Modified Ashworth Scale (MAS); during intervention, inquire about the patient's feelings every 10 minutes, monitor heart rate and respiration, have a blood pressure monitor and pulse oximeter ready; after intervention, record patient reactions.
- Rehabilitation Assessor: Assessments using fNIRS, three-dimensional gait analysis, EEG, sEMG, and MEP were conducted at three time points: baseline (T0), 2-week mid-intervention (T1), and immediately post-intervention at 4 weeks (T2). The FMA-LE and MBI were administered at T0, T1, T2, and at the 6-week follow-up (T3). Safety was evaluated by monitoring changes in all collected data, with any anomalies promptly reported to the safety monitor.

**7.1.2 Monitoring Content and Frequency**

- Vital Signs: Monitor heart rate and respiration every 10 minutes during robot-assisted training; measure blood pressure and heart rate before TMS intervention, monitor appropriately during intervention.
- Symptoms and Signs: Check patients for adverse symptoms such as hearing problems, local pain, and changes in limb function before and after each intervention; comprehensive assessment by the rehabilitation assessor at each assessment time point.
- Equipment: Check the TMS equipment cooling system, navigation calibration, and parameter settings before use; check robot battery, joint range of motion, and emergency stop function; pay attention to equipment operation during use, stop immediately if abnormal; record maintenance status after use; TMS equipment calibrated by the manufacturer every 6 months, robot calibrated annually.

**7.2 Adverse Event Handling**

**7.2.1 Definition of Adverse Events**

Refers to any unintended physical or psychological discomfort experienced by the patient during the study, regardless of its relationship to the intervention, including but not limited to hearing problems, local pain, muscle twitching, joint soreness, muscle fatigue, dizziness, seizures.

**7.2.2 Handling Procedures for Different Types of Adverse Events**

- Seizure: Immediately stop the intervention, lay the patient flat, turn head to one side to keep the airway clear, prevent injury; call for emergency help, monitor vital signs and record the seizure situation; if convulsions last more than 5 minutes or occur consecutively, administer antiepileptic drugs (e.g., diazepam) according to emergency guidelines; assist with further examination after condition stabilizes; report to the Ethics Committee and other relevant departments within 24 hours.
- Hearing Problems: Stop TMS intervention, let the patient rest in a quiet environment; check device sound output, refer to ENT if symptoms do not alleviate.
- Local Pain, Muscle Twitching, Joint Soreness, Muscle Fatigue: Reduce intervention intensity or suspend intervention, let the patient rest, apply cold/heat therapy as appropriate; adjust subsequent intervention parameters based on patient tolerance.
- Dizziness: Stop intervention, assist the patient to lie flat or sit quietly; measure blood pressure and heart rate, arrange further examination if symptoms do not alleviate or worsen.

**7.2.3 Adverse Event Recording and Reporting**

- Recording: After each intervention, promptly record the time of adverse event occurrence, manifestations, related intervention circumstances, handling measures, and outcomes; data entered into the Case Report Form (CRF) and electronic database, key data double-entered, randomly reviewed by a third person.
- Reporting: Report serious adverse events (e.g., seizures) to the Yancheng First People's Hospital Ethics Committee, etc., within 24 hours; mild and moderate adverse events summarized and reported weekly in meetings.

**7.3 Patient Withdrawal Handling**

Perform unblinding for withdrawn patients; make every effort to collect the patient's last medical assessment data and basic information; inform the patient that necessary medical consultation and support are still available after withdrawal.
